# Comprehensive analysis of the *Lycopodium japonicum* mitogenome reveals abundant tRNA genes and *cis*-spliced introns in Lycopodiaceae species

**DOI:** 10.3389/fpls.2024.1446015

**Published:** 2024-08-20

**Authors:** Ning Sun, Fuchuan Han, Suyan Wang, Fei Shen, Wei Liu, Weishu Fan, Changwei Bi

**Affiliations:** ^1^ College of Information Science and Technology and Artificial Intelligence, Nanjing Forestry University, Nanjing, China; ^2^ Research Institute of Subtropical Forestry, Chinese Academy of Forestry, Hangzhou, China; ^3^ Beijing Key Laboratory of Agricultural Genetic Resources and Biotechnology, Institute of Biotechnology, Beijing Academy of Agriculture and Forestry Sciences, Beijing, China; ^4^ College of Optical, Mechanical and Electrical Engineering, Zhejiang A&F University, Hangzhou, China; ^5^ Germplasm Bank of Wild Species, Kunming Institute of Botany, Chinese Academy of Sciences, Kunming, China; ^6^ New Cornerstone Science Laboratory, Institute of Genetics and Developmental Biology, Chinese Academy of Sciences, Beijing, China; ^7^ State Key Laboratory of Tree Genetics and Breeding, Co-Innovation Center for Sustainable Forestry in Southern China, Key Laboratory of Tree Genetics and Biotechnology of Educational Department of China, Key Laboratory of Tree Genetics and Silvicultural Sciences of Jiangsu Province, Nanjing Forestry University, Nanjing, China

**Keywords:** *Lycopodium japonicum*, mitogenome, comparative analysis, RNA editing events, phylogenetic analysis

## Abstract

Lycophytes and ferns represent one of the earliest-diverging lineages of vascular plants, with the Lycopodiaceae family constituting the basal clade among lycophytes. In this research, we successfully assembled and annotated the complete *Lycopodium japonicum* Thunb. (*L*. *japonicum*) mitochondrial genome (mitogenome) utilizing PacBio HiFi sequencing data, resulting in a single circular molecule with a size of 454,458 bp. 64 unique genes were annotated altogether, including 34 protein-coding genes, 27 tRNAs and 3 rRNAs. It also contains 32 group II introns, all of which undergo *cis*-splicing. We identified 195 simple sequence repeats, 1,948 dispersed repeats, and 92 tandem repeats in the *L*. *japonicum* mitogenome. Collinear analysis indicated that the mitogenomes of Lycopodiaceae are remarkably conserved compared to those of other vascular plants. We totally identified 326 RNA editing sites in 31 unique protein-coding genes with 299 sites converting cytosine to uracil and 27 sites the reverse. Notably, the *L. japonicum* mitogenome has small amounts foreign DNA from plastid or nuclear origin, accounting for only 2.81% of the mitogenome. The maximum likelihood phylogenetic analysis based on 23 diverse land plant mitogenomes and plastid genomes supports the basal position of lycophytes within vascular plants and they form a sister clade to all other vascular lineages, which is consistent with the PPG I classification system. As the first reported mitogenome of Lycopodioideae subfamily, this study enriches our understanding of *Lycopodium* mitogenomes, and sets the stage for future research on mitochondrial diversity and evolution within the lycophytes and ferns.

## 1 Introduction

Mitochondria have evolved from independent bacteria into semiautonomous organelles since mitochondrial endosymbiosis and they play significant roles in respiration and metabolism (Gray et al., 1999; Sloan et al., 2018; Bi et al., 2022; Lian et al., 2024; Wang et al., 2024). Furthermore, the mitochondrial genomes (mitogenomes) of plants are crucial for investigating cytoplasmic male sterility mechanisms and molecular breeding (Xiao et al., 2020; Han et al., 2022; Kuwabara et al., 2022; Zhou et al., 2024). In comparison to the relatively compact animal mitogenomes (10-20 kb) and the more conserved plant plastid genomes (plastomes, 100-200 kb) (Wu et al., 2020; Liu et al., 2022; Yang et al., 2023), the plant mitogenome size is extremely variable, ranging from 66 kb in *Viscum scurruloideum* to 11.7 Mb in *Larix sibirica* (Skippington et al., 2015; Putintseva et al., 2020). Furthermore, plant mitogenomes are distinguished by several unique features, including a low gene density, frequent RNA editing events, high intron density within genes, gene transfer and loss, and structural variations between species (Bi et al., 2024b; Palmer and Herbon, 1988; Rice et al., 2013; Jiang et al., 2023; Gong et al., 2024). More than 16,000 complete plastomes have been reported so far (https://ngdc.cncb.ac.cn/cgir). In contrast, the number of complete mitogenomes is less than 700 in the NCBI Nucleotide database (last access January 13th, 2024) (Shen et al., 2022; Wang et al., 2024). The assembly of complete plant mitogenomes presents significantly complex challenges, mainly due to the huge structural variation, long repetitive sequences (repeats) and occurrences of nuclear mitochondrial DNA transfer and mitochondrial plastid DNA transfer.

Previous studies indicated that lycophytes are sister groups to all other vascular plants, rendering them a perfect group for exploring the evolutionary history of vascular plants (Duff and Nickrent, 1999; Pryer et al., 2001; Shen et al., 2018). Lycopodiaceae plants are identified as the basalmost branch among the three lineages within lycophytes (Qiu et al., 2006), making them an ideal option for tracing the most ancient mitogenome of extant vascular plants. The lineages present in lycophytes are three in number: Lycopodiaceae, Selaginellaceae, and Isoetaceae (Øllgaard, 1990; Troia et al., 2016; Wu et al., 2017). To date, only four complete mitogenomes of lycophytes have been released, including *Selaginella moellendorffii* (*S. moellendorffii*), *Isoetes engelmannii* (*I. engelmannii*) and two Lycopodiaceae species: *Phlegmariurus squarrosus* (*P. squarrosus*) and *Huperzia crispata* (*H. crispata*) (Grewe et al., 2009; Hecht et al., 2011; Liu et al., 2012; Cao et al., 2023).

The four existing mitogenomes have yielded significant insights into the degree of mitochondrial genomic diversity across these lineages. For instance, the group I and group II introns were found in *S. moellendorffii* and *I. engelmannii*, several of which are *trans*-splicing introns, while the introns in the mitogenomes of *P. squarrosus* and *H. crispata* are *cis*-splicing and all belong to group II introns. Although our understanding of the phylogenetic relationships of lycophytes and ferns has developed, related studies are still lacking compared with that of seed plants. Consequently, it is urgent to sequence the complete mitogenome from additional Lycopodiaceae members to elucidate the evolutionary status of basal vascular plants and clarify the phylogenetic relationships with the Lycopodiaceae species.

In this research, we conducted the assembly and annotation of the complete *Lycopodium japonicum* Thunb. (*L. japonicum*) mitogenome, which belongs to Lycopodioideae subfamily. The genome composition, intron content, repeats, RNA editing sites, as well as mitochondrial plastid DNA (MIPT) (Mower et al., 2012) of *L*. *japonicum* were analyzed, filling a gap of the mitogenome data in *Lycopodium*. Furthermore, we analyzed the mitogenome using a comparative genomics approach and reconstructed the phylogenetic trees according to protein-coding genes (PCGs). These findings will offer valuable insights for the molecular classification, identification, and germplasm conservation of Lycopodioideae plants and enhance our understanding of the evolutionary significance of lycophytes.

## 2 Materials and methods

### 2.1 Plant materials and sequencing

We gathered fresh leaves of *L*. *japonicum* from Kunming Institute of Botany, Chinese Academy of Sciences and stored them at -80°C for subsequent use. We utilized Hi-DNA secure Plant Kit method to extract High-quality total genomic DNA. The quality and purity were assessed using a Nanodrop 2000 spectrophotometer (ThermoFisher) and a 1.0% agarose gel electrophoresis. Subsequently, we constructed sequencing libraries using the high-integrity genomic DNA and the SMRTbell Express Template Prep Kit 2.0 (PacBio Biosciences, CA, USA). Ultimately, the HiFi sequencing data was generated using the PacBio Revio platform.

### 2.2 Assembly and annotation of the *L*. *japonicum* mitogenome

The HiFi sequencing data was fed into PMAT v1.31 (Bi et al., 2024a) to assemble the mitogenome of *L. japonicum*. The parameters and mode used for PMAT were ‘-st hifi -g 2.3G -cpu 50’ and ‘autoMito’ mode, respectively. The genome size of *L. japonicum* was estimated using *Lycopodium clavatum* as the reference (Yu et al., 2023). Bandage was then used to visualize and disentangle the raw assembly graph of *L. japonicum* mitogenome (Wick et al., 2015). Online program IPMGA (http://www.1kmpg.cn/ipmga/) was used to annotate the *L. japonicum* mitogenome. Further, tRNAs and rRNAs were verified by BLASTn and tRNAscan-SE v2.0, respectively (Camacho et al., 2009; Chan et al., 2021). Afterwards, the introns were designated by their orthologous gene positions within *Marchantia polymorpha* mitogenome (Dombrovska and Qiu, 2004). All the annotations were manually checked and reviewed using MacVector v18.5. The mitogenome map and the intron contents of the *L*. *japonicum* was visualized using PMGmap with default parameters (Zhang et al., 2024).

### 2.3 Identification of repeat elements in the *L*. *japonicum* mitogenome

Using online platform MISA (https://webblast.ipk-gatersleben.de/misa/), we identified simple sequence repeats (SSRs) present in the *L. japonicum* mitogenome (Beier et al., 2017). The thresholds for the minimum numbers of repetitions for mononucleotides, dinucleotides, trinucleotides, tetranucleotides, pentanucleotides, and hexanucleotides were set as 10, 5, 4, 3, 3, and 3 respectively. Another online program REputer (https://bibiserv.cebitec.uni-bielefeld.de/reputer) was utilized to detect forward, reverse, palindromic and complement dispersed repeats (Kurtz et al., 2001). The parameters for the maximum number of computed repeats, minimal repeat size, and hamming distance were individually configured to 5000, 30, and 3, separately. Furthermore, we detected the tandem repeats of the mitogenome with Tandem Repeats Finder v4.09 (https://tandem.bu.edu/trf/trf.html) with default parameters (Benson, 1999). All repeat elements were manually checked and finally visualized utilizing TBtools v1.132 with ‘Advanced Circos’ module (Chen et al., 2020).

### 2.4 Detection of RNA editing events in *L. japonicum* mitogenome

RNA editing, referring to the insertion, deletion, or substitution of bases in genomic transcripts, plays significant roles in increasing the diversity of gene transcription and function (Fan et al., 2019). LncRNA sequencing data were generated from the same sample of *L. japonicum* to detect the RNA editing events in the *L*. *japonicum* mitogenome. We extracted coding sequences of each PCG with 100 bp upstream and downstream to construct reference sequences (Yang et al., 2023). Next, we used BWA to map strand-specific RNA sequencing data to reference sequences (Li and Durbin, 2009), with parameter settings ‘mem -t 40 -k 55 -d 150 -r 1.0 -M’. Sambamba was used to sort BAM files and to filter secondary or supplementary alignments (Tarasov et al., 2015). REDItools v2 was utilized to detect the RNA editing sites according to mapping results (Picardi and Pesole, 2013). We employed minimap2 to align the HiFi sequencing data to the *L*. *japonicum* mitogenome (Li, 2018; 2021), and subsequently extracted sites that were not recognized as SNPs, which indicated potential RNA editing locations. IGV program was finally used to manually verify and validate all possible RNA editing sites (Robinson et al., 2011; Kim et al., 2019). Subsequently, we also used HISAT2 to identify RNA editing events and compared the results with those of REDItools.

### 2.5 Whole-genome collinearity analysis

To compare the mitogenome structure of *L*. *japonicum* within Lycopodiaceae and other land plants, we downloaded seven mitogenomes from NCBI, including *P*. *squarrosa* (NC_017755), *H. crispata* (NC_071971), *Marchantia paleacea* (NC_001660), *Ophioglossum vulgatum* (NC_065260), *Ginkgo biloba* (NC_027976), *Oryza sativa* (NC_007886), and *Arabidopsis thaliana* (NC_037304). All mitogenomes were aligned against the *L*. *japonicum* mitogenome using BLASTn. Only collinear results with the minimum alignment length ≥200 bp and the minimum identity ≥80% were selected for further analysis. The NGenomeSyn program was used to perform and visualize the collinearity analysis (He et al., 2023).

### 2.6 Analysis of MIPTs

Higher plant mitogenomes have numerous sequences migrating from their plastomes. To detect the MIPTs of *L. japonicum*, we assembled the complete *L. japonicum* plastome (accession number: NC_085262). Subsequently, we employed BLASTn to detect the homologous fragments between the mitogenome and plastome with parameters ‘-word_size 9 -evalue 1e-5 -reward 2 -gapopen 5-gapextend 2 -penalty -3 -outfmt 6’. The results with lengths ≥50 bp and matching rate ≥70% were selected for further analysis and were manually annotated to check the gene location in the MIPTs. Ultimately, we visualized the MIPT results utilizing TBtools v1.132 with ‘Advanced Circos’ module.

### 2.7 Phylogenetic analysis

To construct the phylogenetic trees for mitochondria and plastids, we downloaded mitogenomes and plastomes of another 22 species from NCBI Nucleotide database. *Saccharina japonica* and *Laminaria rodriguezii* were set as outgroup. We selected 15 conserved PCGs of mitogenomes (*atp6*, *atp9*, *cox1*, *cox2*, *cox3*, *cob*, *mttB*, *nad1*, *nad2*, *nad3*, *nad4*, *nad4L*, *nad5*, *nad6*, and *nad9*) and 26 conserved PCGs of plastomes (*atpA*, *atpB*, *atpH*, *atpI*, *petA*, *petD*, *psaA*, *psaB*, *psaC*, *psbA*, *psbB*, *psbC*, *psbD*, *psbE*, *psbH*, *rbcL*, *rpl2*, *rpl14*, *rpl16*, *rpl20*, *rpl33*, *rpoC2*, *rps3*, *rps4*, *rps7*, and *rps11*) for multiple sequence alignment with MAFFT v7.407 (Rozewicki et al., 2019). Subsequently, we concatenated the aligned sequences to reconstruct the maximum likelihood (ML) tree using IQ-TREE v2.0.3 with a bootstrap value of 1000 replicates (Minh et al., 2020). ‘GTR+F+I+G4’ was chosen as the best fit evolutionary model of mitochondrial and plastid ML trees based on Bayesian Information Criterion (BIC) scores. Eventually, the ML tree results were visualized by the online program iTOL (https://itol.embl.de/) (Letunic and Bork, 2021).

## 3 Results

### 3.1 Characteristics of the *L*. *japonicum* mitogenome

**Figure 1:**
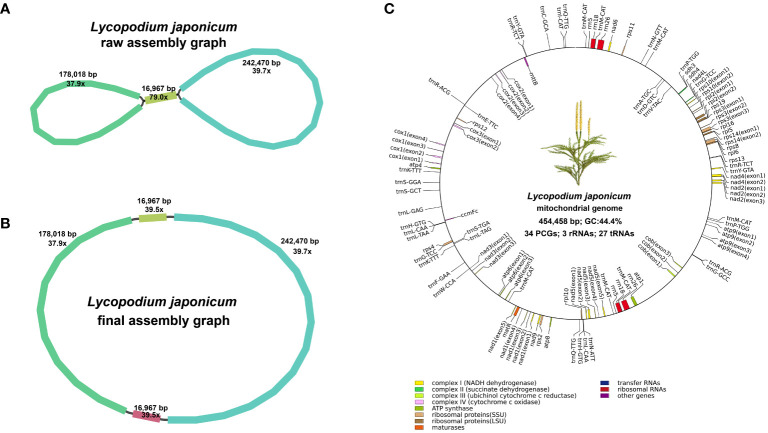
Assembly graphs and genome map of the mitogenome. **(A)** Raw assembly graph. **(B)** Disentangled assembly graph. **(C)** The genome map of the *Lycopodium japonicum* mitogenome. Different types of genes are depicted using different color blocks.

Utilizing Bandage for contig visualization, and after excluding duplicated regions, we delineated a single, putative circular chromosome for *L*. *japonicum* mitogenome (**Figures 1A, B**). The mitogenome of *L*. *japonicum* has a total length of 454,458 bp and a GC content of 44.4%. We detected 64 unique genes in *L*. *japonicum* mitogenome, with the coding regions comprising 8.82% (40,083 bp) of the mitogenome, including 34 PCGs (26,844 bp, 5.91%), 3 rRNAs (10,054 bp, 2.21%) and 27 tRNAs (3,185 bp, 0.70%), respectively (**Figure 1C**; **Supplementary Table S1**). Among the PCGs, we identified 18 core mitochondrial genes (*atp1*, *atp4*, *atp6*, *atp9*, *cob*, *cox1*, *cox2*, *cox3*, *matR*, *mttB*, *nad1*, *nad2*, *nad3*, *nad4*, *nad4L*, *nad5*, *nad6*, and *nad9*) and 16 variable genes (*rpl2*, *rpl5*, *rpl6*, *rpl10*, *rpl16*, *rps2*, *rps3*, *rps4*, *rps10*, *rps11*, *rps12*, *rps13*, *rps14*, *rps19*, *sdh3* and *sdh4*), while *atp8*, *rps8* and *ccmFc* was annotated as a pseudogene. Notably, three cytochrome c biogenesis (*ccm*) genes (*ccmB*, *ccmC*, and *ccmFn*) and *nad7* were absent. In addition, each of the three rRNA genes is present in two copies while one tRNA gene (*trnM*-CAT) had seven copies. We have deposited the complete *L*. *japonicum* mitogenome in NCBI Nucleotide database under the accession number NC_080981. Furtherly, we compared the gene content with other land plants mitogenomes (**Supplementary Figure S1A**). The results showed that all *ccm* genes are absent or pseudogenes in the mitogenomes of *Ophioglossum vulgatum*, *Haplopteris ensiformis* and all lycophytes, but exist in *P*. *nudum*, all gymnosperms and all angiosperms.

Across the 34 PCGs, we observed three distinct start codons and five different stop codons. The majority of PCGs initiate with ATG, while *cox1*, *nad1*, *nad5*, *nad9*, *rpl13*, and *sdh4* employ ACG as their start codon. The stop codons TAA (16 genes: *atp1*, *atp4*, *atp6*, *nad2*, *nad3*, *nad4*, *nad4L*, *nad5*, *nad9*, *rpl16*, *rps4*, *rps10*, *rps11*, *rps12*, *sdh3*, and *sdh4*), CAA (three genes: *atp9*, *cox3*, and *rpl6*), TGA (four genes: *cob*, *nad6*, *rps3*, and *rps13*), TAG (nine genes: *cox1*, *matR*, *mttB*, *rpl2*, *rpl5*, *rpl10*, *rps2*, *rps14*, and *rps19*), CGA (two genes: *cox2* and *nad1*) were identified.

We detected 32 introns within 15 PCGs (*atp6*, *atp9*, *cob*, *cox1*, *cox2*, *cox3*, *nad1*, *nad2*, *nad3*, *nad4*, *nad5*, *rpl2*, *rps3*, *rps10*, and *rps14*), and all introns are categorized as *cis*-splicing introns (**Supplementary Figure S2**). Mitochondrial introns are classified into two types for the majority of eukaryotes, group I and group II, based on the mechanisms by which they splice and the secondary structures (Michel and Westhof, 1990; Saldanha et al., 1993). In the mitogenome of *L. japonicum*, all 32 introns are group II introns. To elucidate the relatively conserved pattern of intron evolution, we compared *cis*- and *trans*-spliced intron content in mitogenomes from bryophyte to seed plants. As shown in **Supplementary Figure S1B**, *P. squarrosus* and *L. japonicum* share 32 identical group II introns, with *H*. *crispata* containing 31 group II introns and only missing rps10i235. In the mitogenomes of five lycophytes, certain introns such as atp6i439, atp9i21, atp9i87, cobi693, and cobi787 are present, yet these are conspicuously absent in many other vascular plants. Among the 21 species examined, group I introns were exclusively found in the mitogenomes of *I*. *engelmannii*, *S*. *moellendorffii*, *Psilotum nudum*, and three bryophytes, but were notably absent from the mitogenomes of other vascular plants (**Supplementary Figure S1C**).

### 3.2 Analysis of repeats and MIPTs

**Figure 2:**
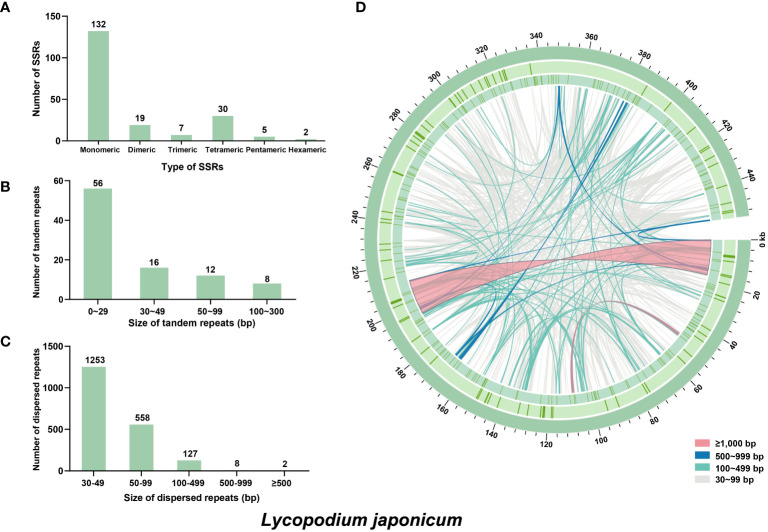
Repeat elements identified in the *Lycopodium japonicum* mitogenome. **(A)** The frequency of identified SSRs. **(B)** The frequency of identified tandem repeats. **(C)** The frequency of identified dispersed repeats. **(D)** Distribution of repeat elements in the mitogenome. The outer circle represents the tandem repeats, followed by the SSRs, and the inner lines represent the dispersed repeats. Detailed information of dispersed and tandem repeats is shown in **Supplementary Tables S2** and **S3**, respectively.

In mitochondrial DNA, the dynamic restructuring of its architecture was largely mediated by the presence of repeats, including SSRs, dispersed repeats and tandem repeats. A total of 195 SSRs were detected in *L. japonicum* using web-based platform MISA, which were composed of 132 monomeric, 19 dimeric, 7 trimeric, 30 tetrameric, 5 pentameric, and 2 hexameric repeats (**Figure 2A**). It was observed that the repeat units of A/T (94 repeats), AT/AT (nine repeats), AAT/ATT (five repeats), and AAAG/CTTT (eight repeats) were more prevalent in the monomeric, dimeric, trimeric, and tetrameric categories, respectively. Additionally, 92 tandem repeats were identified (**Figure 2B**), with nearly half of these ranging between 50 and 99 bp in length, while only two exceeded 500 bp (651 bp and 650 bp, respectively). We identified 1,949 dispersed repeats in *L. japonicum* mitogenome with length ≥30 bp (total length: 124,620 bp, account for 27.42% of the whole mitogenome), including 1,001 forward repeats, 943 palindromic repeats, and only five reverse repeats. The majority of the dispersed repeats are shorter than 100 bp, with a count of 1,811 repeats, and only 10 repeats exceed 500 bp, with the maximum length being 16,967 bp (**Figures 2C, D**).

**Figure 3:**
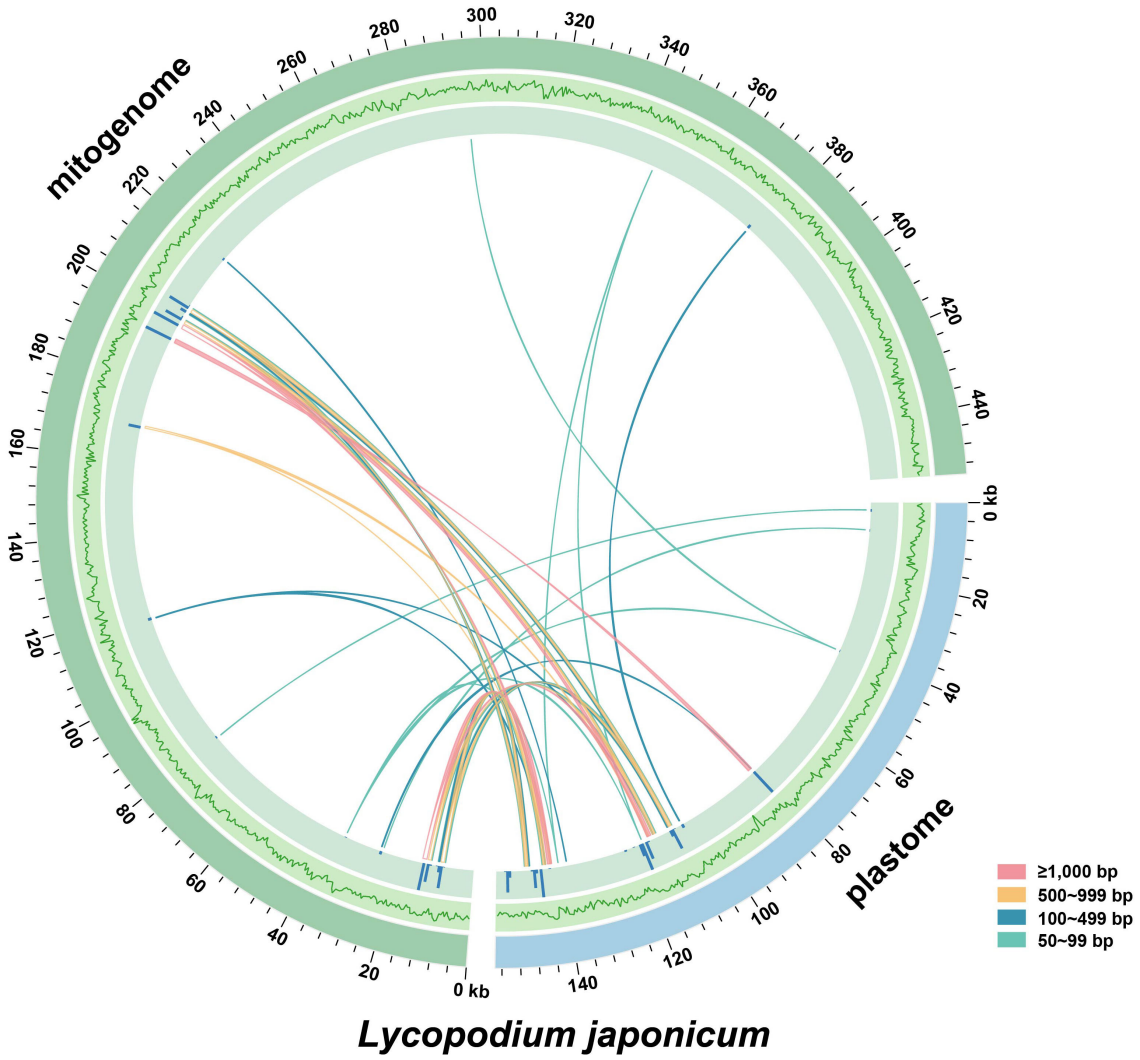
Distribution of MIPTs in *Lycopodium japonicum*. The blue and green arcs represent the plastid and mitochondrial genomes of *L. japonicum*, respectively. The outer circle represents the GC content of the two genomes, followed by the bars representing the length of MIPTs. Lines between the two arcs represent the MIPTs. Detailed information of MIPTs is shown in **Supplementary Table S4**.

The mitogenomes of higher plants contain numerous sequences that migrated from nuclear and plastid genomes (Mower et al., 2012). In this study, we identified 39 MIPTs (12,767 bp in length) with a similarity threshold of 70%, which represent 8.40% of the plastome and 2.81% of the mitogenome, respectively (**Figure 3**). Five MIPTs are more than 1,000 bp in length, with four of them uniformly measuring 1,165 bp. However, among these homologous sequences, only partial plastid genes are annotated locating within the MIPTs, including *atpA*, *ndhF*, *rps11*, *rrn16S*, *rrn23S*, *trnN-GUU*, *and trnF-GAA*.

### 3.3 Analysis of whole-genome collinearity

**Figure 4:**
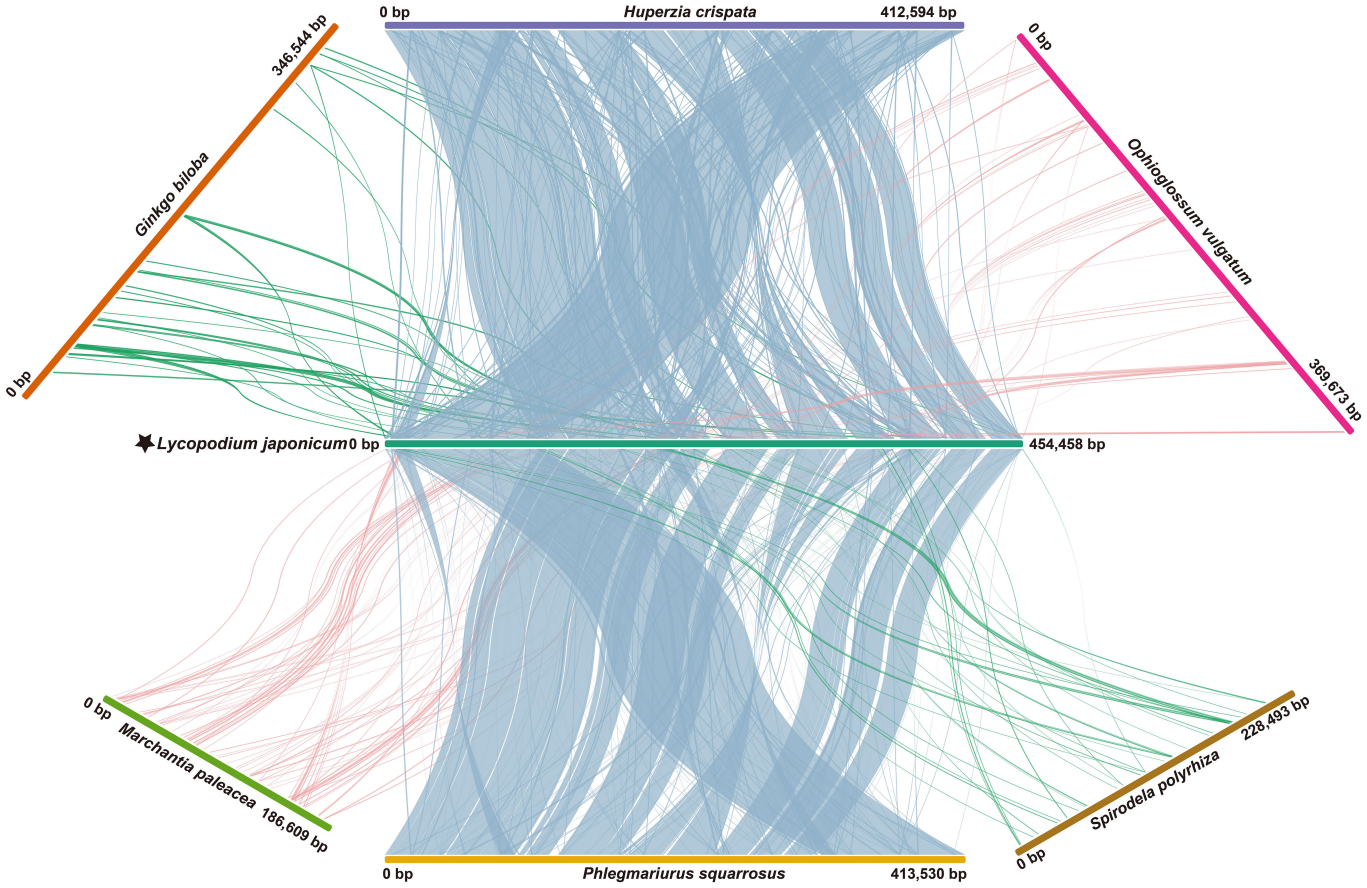
Whole mitogenome collinearity analysis. Long strips of different colors represent different plant mitogenomes. Linear blocks represent collinear segments. Species names and IDs used in collinearity analysis are shown in **Supplementary Table S7**.

We conducted a comparative study to analyze the mitogenome collinear regions within selected Lycopodiaceae and within land plants representatives. When comparing the mitogenomes of *L. japonicum* and *H. crispata*, we identified 252 locally collinear blocks (LCBs), with total size of 363,943 bp, which represents 88.21% of the entire mitogenome of *H. crispata*. A comparison with *P. squarrosa* revealed 231 LCBs (total length: 395,656 bp), which constitutes 95.68% of the mitogenome of *P. squarrosa* (**Figure 4**). It is observed that compared with other land plants mitogenomes, the number of LCBs, average length and collinear identity score, were consistently lower than those observed in *P. squarrosa* and *H. crispata*. Additionally, numerous sequence rearrangements were identified among the *L. japonicum* mitogenome and those of other species.

### 3.4 Analysis of RNA editing events

**Figure 5:**
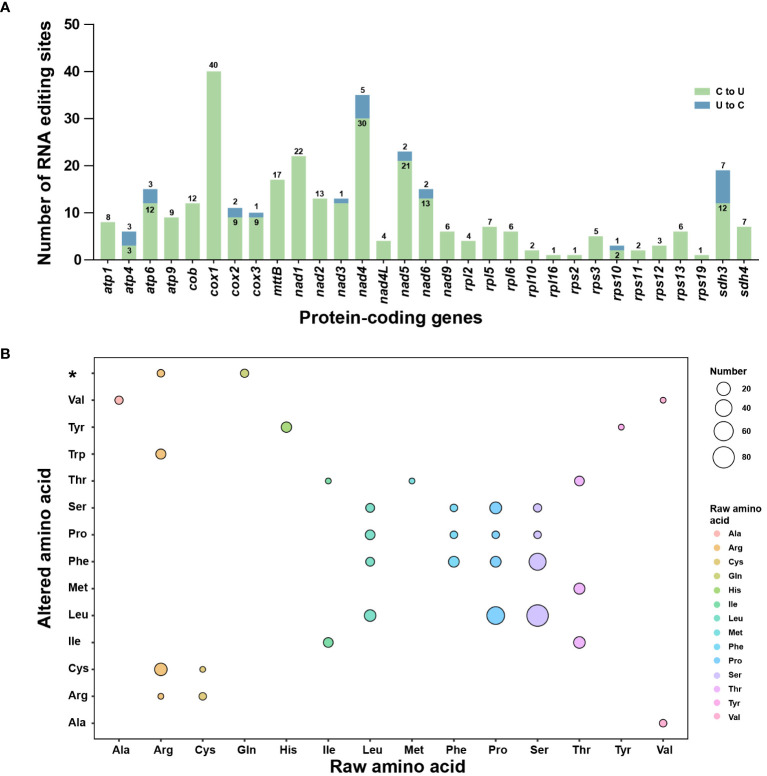
RNA editing events in the *Lycopodium japonicum* mitogenome. **(A)** Characteristic of RNA editing sites across all PCGs in the mitogenome. The green bars indicate C-U RNA editing sites while the green bars are U-C RNA editing sites. The quantity of both is marked above or inside the bar. **(B)** The synonymous and non-synonymous substitutions caused by RNA editing. The color and size of the circle represent the type of raw amino acid and the number of the substitution, separately. The asterisk on the vertical axis represents stop codons. Detailed information of RNA editing sites is shown in **Supplementary Table S5**.

To estimate the extent of RNA editing sites in *L. japonicum* mitogenome, the BWA program was employed to identify RNA editing sites based on 14.57 Gb RNA sequencing data. After manually checking using IGV and comparison with SNPs, we finally identified 326 RNA editing sites among 31 PCGs of *L. japonicum* mitogenome. Notably, most of these sites (299, accounting for 91.72%) involved C-U substitutions, while 27 sites (8.28%) exhibited U-C substitutions (**Supplementary Table S5**). Specifically, 21 genes (*atp1*, *atp9*, *cob*, *cox1*, *mttB*, *nad1*, *nad2*, *nad4L*, *nad9*, *rpl2*, *rpl5*, *rpl6*, *rpl10*, *rpl16*, *rps11*, *rps2*, *rps3*, *rps12*, *rps13*, *rps19*, and *sdh4*) exclusively contain C-U RNA editing sites, while 10 genes (*atp4*, *atp6*, *cox2*, *cox3*, *nad3*, *nad4*, *nad5*, *nad6*, *rps10*, and *sdh3*) exhibit both C-U and U-C RNA editing sites, with *cox1* containing the highest number of RNA editing sites, totaling 40 (**Figure 5A**). Notably, three genes (*matR*, *rps4*, and *rps14*) were found to lack any RNA editing sites. Out of the total RNA editing sites detected, 79 (24.23%) sites were located in the first codon position, 204 (62.58%) in the second codon position, and 43 (13.19%) in the third codon position. We observed that RNA editing events had reconstituted six start codons and five stop codons in 11 PCGs (**Supplementary Table S1**). Subsequently, we analyzed the synonymous and non-synonymous substitutions caused by RNA editing events (**Figure 5B**). We detected 33 substitution types (10 kinds of synonymous and 23 kinds of non-synonymous substitutions) in the *L. japonicum* mitogenome. The most frequent substitution involved the transformation of 82 amino acids from Ser to Leu.

### 3.5 Phylogenetic analysis

**Figure 6:**
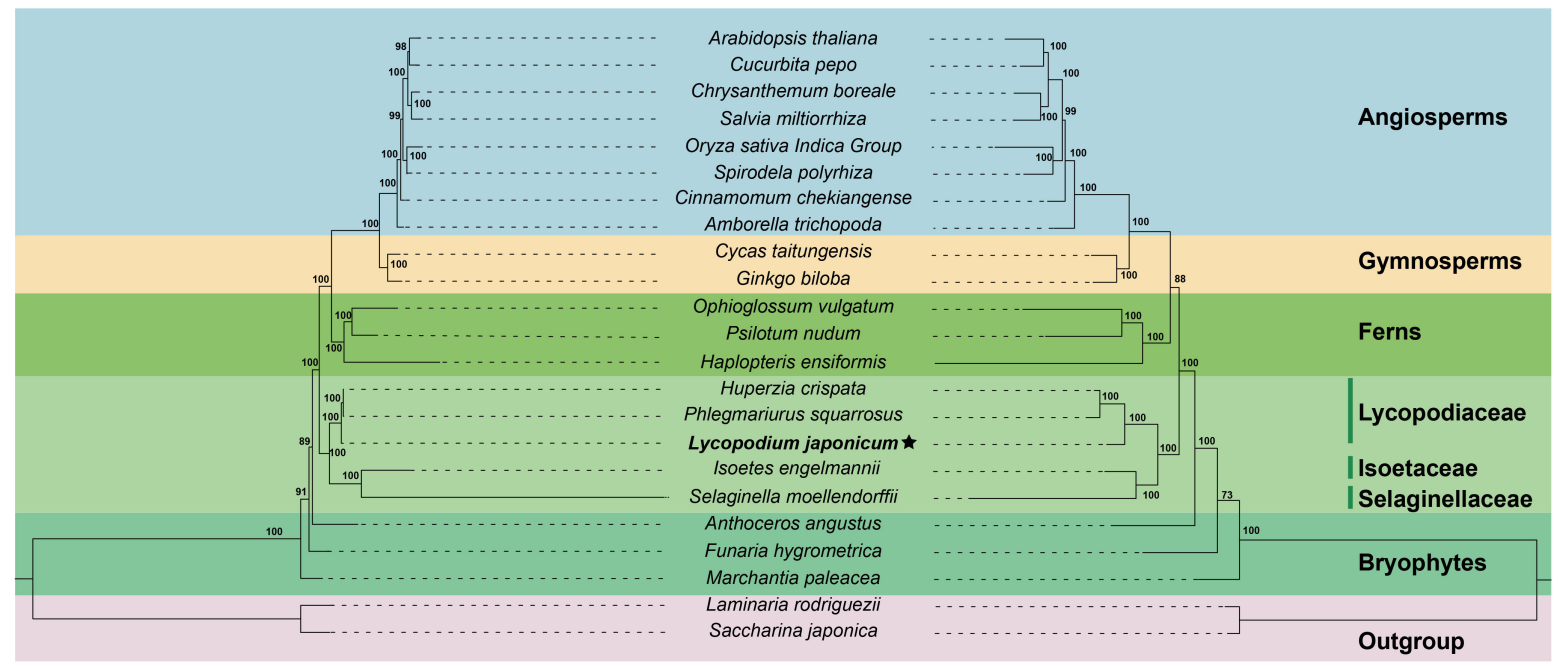
The ML trees of 23 plant species based on 15 mitochondrial PCGs (left) and complete plastome sequences (right). The *Lycopodium japonicum* mitogenome is bold and labeled with an asterisk. *Saccharina japonica* and *Laminaria rodriguezii* were selected as outgroup. Bootstrap values are marked on each branch. Colors indicate the groups for each species. Plant names and NCBI accession numbers used in the phylogenetic analysis are provided in **Supplementary Table S7**.

The ML tree was constructed based on 15 conserved mitochondrial PCGs from 23 plant species to investigate the phylogenetic position of *L*. *japonicum*. We selected two algal species as outgroups. The phylogenetic analysis showed that *L*. *japonicum* clustered together with the other two Lycopodiaceae species: *P. squarrosus* and *H. crispata* (**Figure 6**). The three Lycopodiaceae species are a sister group of *S. moellendorffii* + *I. engelmannii*. The reconstructed ML tree demonstrates a well-supported phylogeny of lycophytes (bootstrap values = 100), and the entire topology displays a high degree of concordance with the PPG I classification system (PPG I, 2016). We further validated our phylogeny by reconstructing the phylogenetic tree using plastome sequences. The structural comparison of the ML trees derived from sequences of mitogenomes and plastomes reveals a perfect consistency, validating the precision of our phylogenetic analyses.

## 4 Discussion

### 4.1 The moderate size of the *L*. *japonicum* mitogenome

Previous studies that put forward the hypothesis that the typical size of the vascular plant mitogenome is around 400 kb (Guo et al., 2017). In this study, the mitogenome of *L*. *japonicum* is structured as a circular DNA molecule with a GC content of 44.4% and a size of 454,458 bp. The mitogenomes of three Lycopodiaceae species (*L. japonicum*, *P*. *squarrosus*, and *H*. *crispata*) are relatively conserved. Each of these mitogenomes exhibits a typical single circular structure and varies slightly in length, ranging from 412,594 bp (*H*. *crispata*) to 454,458 bp (*L. japonicum*). The mitogenomes of ferns, however, exhibit variability in structure, GC content, and length when compared with those of Lycopodiaceae mitogenomes. For instance, the mitogenome size of *Dryopteris crassirhizoma* is 313 kb in length with a GC content of 49.0% (Song et al., 2021). The *Ophioglossum californicum* (*O*. *californicum*) mitogenome exhibits a larger mitogenome of 372 kb and a higher GC content of 52.2% (Guo et al., 2017). The *Psilotum nudum* (*P*. *nudum*) mitogenome is composed of two circular molecules with respective sizes of 264 kb and 364 kb, having a total GC content of 51.2% (Guo et al., 2017; Song et al., 2021).

Studies conducted previously revealed that the mitogenomes of ferns contain abundant repeats, such as *P*. *nudum* (52.7–63.3%) and *O*. *californicum* (37.1–44.3%) (Guo et al., 2017). In comparison to *P*. *nudum* and *O*. *californicum*, the Lycopodiaceae mitogenomes exhibit a notable decrease in the proportion of repeats. Specifically, in the *L. japonicum* mitogenome, repeats make up 27.42% of the entire mitogenome, a proportion comparable to that of *P*. *squarrosus* (26.36%). Additionally, the results of MIPT analysis indicate that the *L. japonicum* mitogenome has limited foreign DNA from plastid or nucleus, which is a characteristic shared with the mitogenomes of *S*. *moellendorffii* and *P*. *squarrosus* (Liu et al., 2012; Wu et al., 2017). However, the foreign DNAs from plastid or nuclear are detected in the mitogenomes of *H*. *crispata*, *I*. *engelmannii*, *Cycas taitungensis* and numerous angiosperms (Troia et al., 2016; Cao et al., 2023). It seems that the mitogenome of *L. japonicum* maintains a stable size, probably because it has not experienced the substantial invasion of foreign DNA from plastid or nucleus, a phenomenon seen in certain angiosperm mitogenomes (Giegé et al., 2008; Babbitt et al., 2015).

### 4.2 The loss of *ccm* genes and abundant tRNAs in the mitogenomes of Lycopodiaceae

*Ccm* genes are typically present in the mitogenomes of most gymnosperms and angiosperms; however, they are absent or present as pseudogenes in all lycophytes mitogenomes. In the *L. japonicum* mitogenome, three *ccm* genes (*ccmB*, *ccmC*, and *ccmFn*) are absent, with only *ccmFc* present as a pseudogene. The loss of *ccm* genes is also found in the mitogenomes of *P*. *squarrosus* and *H*. *crispata* (Liu et al., 2012; Cao et al., 2023). These *ccm* genes are absent in some fern mitogenomes like *Dryopteris crassirhizoma* and *O*. *californicum*, but present in other fern species such as *P*. *nudum* (Troia et al., 2016; Guo et al., 2017; Wu et al., 2017; Song et al., 2021) (**Supplementary Figure S1A**). The aforementioned results suggest that the pathway of cytochrome c maturation seems to have been inherited from the original proto-mitochondrion but it has been lost in Lycopodiaceae species and some ferns (Babbitt et al., 2015).

Protein synthesis in plant mitogenomes necessitates 21 different tRNAs, but some tRNAs are lost frequently during the evolutionary process of higher plant mitogenomes. For example, *S*. *moellendorffii* mitogenome lacks all tRNAs, and *I*. *engelmannii* only maintains 13 intact tRNAs (Troia et al., 2016; Wu et al., 2017). In this study, a total of 27 unique tRNAs were annotated in the mitogenome of *L. japonicum*. Similarly, the mitogenomes of *P*. *squarrosus* and *H*. *crispata* contain 26 and 29 unique tRNAs, respectively (Liu et al., 2012; Cao et al., 2023). The findings suggest that the tRNA content in Lycopodiaceae mitogenomes is much higher than that of other species.

### 4.3 The loss of introns during the evolution of land plants

Studies conducted previously showed that it is challenging to infer the ancestral intron contents of vascular plants due to the significant differences in intron distribution observed among lycophytes, ferns, and seed plants (Guo et al., 2017). In the mitogenome of *L. japonicum*, a total of 32 group II introns were detected, all of which are *cis*-splicing introns. Moreover, within the mitogenomes of Lycopodiaceae species, the number of introns is very abundant, and all these introns undergo *cis*-splicing (**Supplementary Figure S1B**). The results strongly support that the mitogenomes of Lycopodiaceae species exhibit low levels of recombination and are highly conservative. In the lineage of land plants, the mitogenomes contain a high number of group II introns, whereas group I introns are much less common and display an irregular pattern of distribution across different species (Mower, 2020). The evolutionary genesis of the five different group I introns (cox1i375, cox1i395, cox1i624, cox1i876, and cox1i1305) found in either ferns or lycophytes remains ambiguous, given their highly specific distribution patterns within the vascular plant lineage. To elucidate whether the group I introns have been acquired through vertical or horizontal gene transfer, it is essential to undertake a more comprehensive study that includes a wider array of taxa. These results would enhance our understanding of the evolutionary dynamics governing these genetic elements within the plant mitogenomes.

### 4.4 Low RNA editing level in the mitogenomes of Lycopodiaceae species

RNA editing event, which involves the post-transcriptional modification of an RNA sequence through substitutions, deletions, or insertions, contributes significantly to transcriptome diversity (Lukeš et al., 2021). It is likely that RNA editing events take place in the *L*. *japonicum* mitogenome, given that 11 PCGs necessitate the reconstruction of start or stop codons for accurate annotation (**Supplementary Table S1**). In this study, 326 RNA editing sites (299 C-U sites and 27 U-C sites) were detected in the *L. japonicum* mitogenome based on 14.57 Gb lncRNA sequencing data. Previous reports demonstrated that RNA editing events vary widely across lycophytes mitogenomes. Specifically, the amount of RNA editing sites varies among different species, with *P*. *squarrosus* exhibiting a relatively low count of 364 sites, while *I*. *engelmannii* and *S*. *moellendorffii* have significantly higher numbers, with 1,782 and 2,152 sites, respectively (Liu et al., 2012; Troia et al., 2016; Wu et al., 2017). It can be deduced that the mitogenomes of Lycopodiaceae species exhibit low RNA editing level according to the given information. Furthermore, we utilized HISAT2 to validate these RNA editing sites. The findings indicated that HISAT2 and bwa share identical 272 C-U RNA editing sites. It is noteworthy that no U-C editing sites were detected using HISAT2 (**Supplementary Tables S5** and **S6**). Although all RNA editing sites were manually verified using IGV, it is imperative to conduct Sanger sequencing and PCR to acquire a more precise and accurate result.

### 4.5 Phylogenetic analysis of *L*. *japonicum*

Based on PPG I, two pteridophyte classes are recognized, including Lycopodiopsida (lycophytes) and Polypodiopsida (ferns) (PPG I, 2016). The fossil record indicates that lycophytes and ferns are the oldest vascular plants on Earth, dating back to approximately 400 million years ago (Qi et al., 2018; Shen et al., 2018). As a result, the mitogenomes of lycophytes represent the most ancient form of vascular plant mitogenomes. In this study, we reconstructed the phylogenetic trees of 23 plant species based on 15 conserved mitochondrial PCGs and whole plastid genome sequences, separately. Both phylogenetic trees support that *L. japonicum* is a sister group to the clade clustered with *P*. *squarrosus* and *H*. *crispata*, thereby corroborating the familial and generic relationships among the three species. Additionally, the results of phylogenetic analysis also suggest that lycophyte are the basal group of vascular plants, in accordance with the PPG I classification system. Our results conclusively demonstrate that lycophytes are the oldest vascular plants and serve as transitional evolutionary forms bridging the gap between bryophyte and higher vascular plants (Qiu et al., 2006; Guo et al., 2017). Furthermore, the bootstrap values of most nodes are greater than 95%, indicating the robustness and reliability of the recovered phylogeny based on well-conserved mitochondrial PCGs.

## 5 Conclusion

In this study, we successfully assembled the *L. japonicum* mitogenome into a circular molecule with a size of 454,458 bp. We annotated 64 unique genes in the *L*. *japonicum* mitogenome, including 34 PCGs, 27 tRNAs and 3 rRNAs. 32 introns were identified in *L. japonicum* mitogenome and all of them are *cis*-splicing. The MIPTs analysis revealed the absence of any complete plastid genes in the *L. japonicum* mitogenome. Collinearity results indicated that the Lycopodiaceae species mitogenomes are highly conserved in terms of structure and size. Additionally, we detected 326 RNA editing sites in the *L. japonicum* mitogenome, indicating that *L. japonicum* exhibits a low RNA editing level in lycophytes mitogenomes. The reconstructed ML trees of mitogenome and plastome strongly support that *L. japonicum* forms a sister group to the clade containing by *P*. *squarrosus* and *H*. *crispata*, while lycophytes are identified as the basal group of vascular plants. Moreover, we analyzed the repeats of the *L. japonicum* mitogenome and explored the pattern of intron evolution from bryophytes to angiosperms. As the inaugural reported mitogenome in the Lycopodioideae subfamily, this study provides invaluable information for the assembly of other species within the same subfamily and contributes insightful perspectives on intron evolution, RNA editing, phylogeny, and mitochondrial evolution in lycophytes.

## Data Availability

The datasets presented in this study can be found in online repositories. The names of the repository/repositories and accession number(s) can be found below: https://www.ncbi.nlm.nih.gov/, NC_080981 https://www.ncbi.nlm.nih.gov/, NC_085262 https://www.ncbi.nlm.nih.gov/, SRR24785435 https://www.ncbi.nlm.nih.gov/, SRR28249616.

## Supplementary material

Supplementary Figure 1 Gene content and intron content in 21 land plant mitogenomes. **(A)** Gene content. **(B)** Group II introns. **(C)** Group I introns. The *Lycopodium japonicum* mitogenome is bold and labeled with an asterisk. Each intron is named in accordance with its position relative to the homologous gene in the *Marchantia polymorpha* mitogenome. Species names and IDs are shown in **Supplementary Table S7**.

Supplementary Figure 2 The exon and intron contents of the *Lycopodium japonicum* mitogenome.
