# Supplementary figures and images for: Comprehensive analysis of the Lycopodium japonicum mitogenome reveals abundant tRNA genes and cis-spliced introns in Lycopodiaceae species
Source: Front Plant Sci. 2024 Aug 20;15:1446015. doi: 10.3389/fpls.2024.1446015 (PMC11368720; doi:10.3389/fpls.2024.1446015)

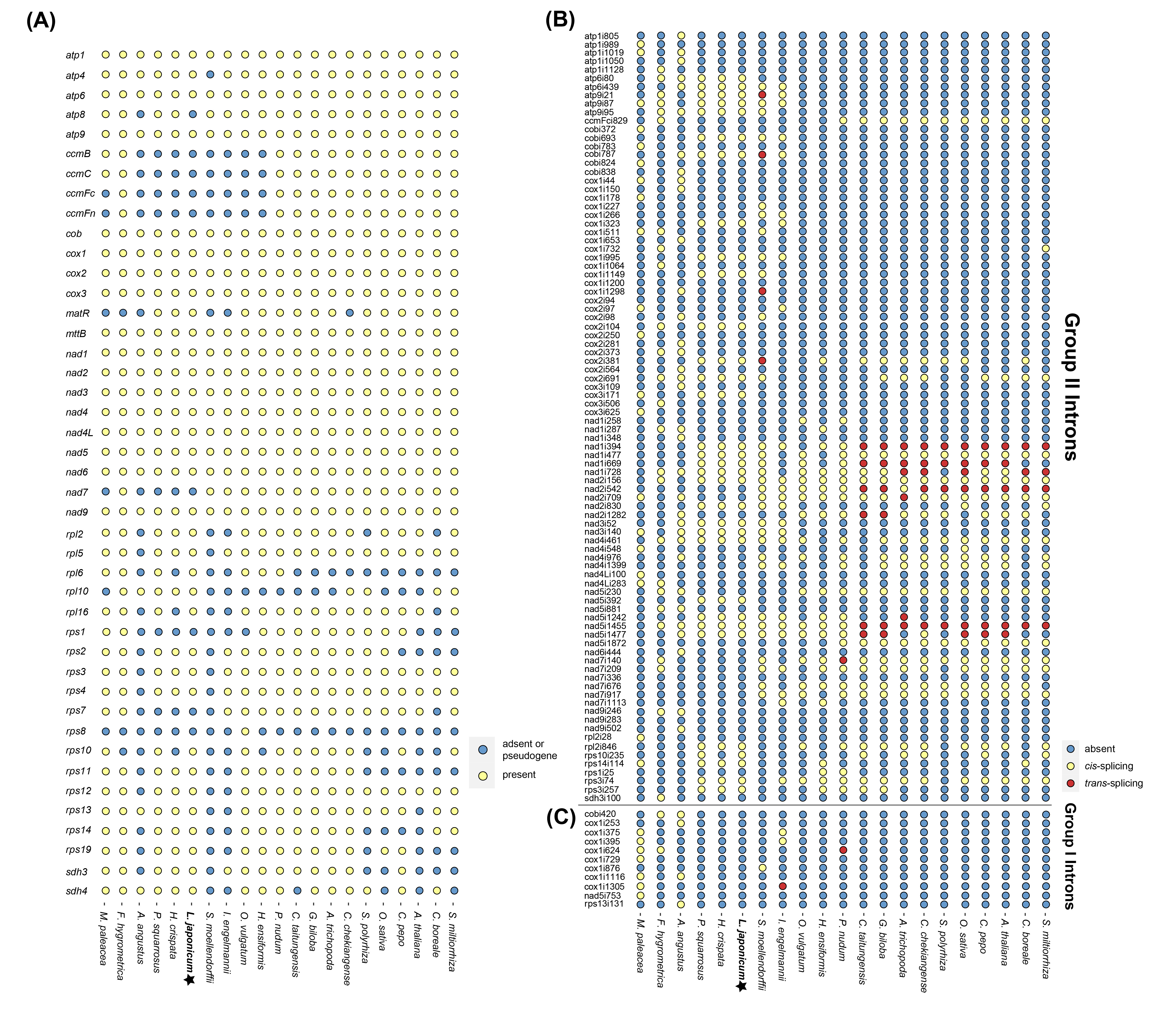

Supplement: Supplementary Figure 1 — Gene content and intron content in 21 land plant mitogenomes. (A) Gene content. (B) Group II introns. (C) Group I introns. The Lycopodium japonicum mitogenome is bold and labeled with an asterisk. Each intron is named in accordance with its position relative to the homologous gene in the Marchantia polymorpha mitogenome. Species names and IDs are shown in Supplementary Table S7 . [file Image1.jpeg]

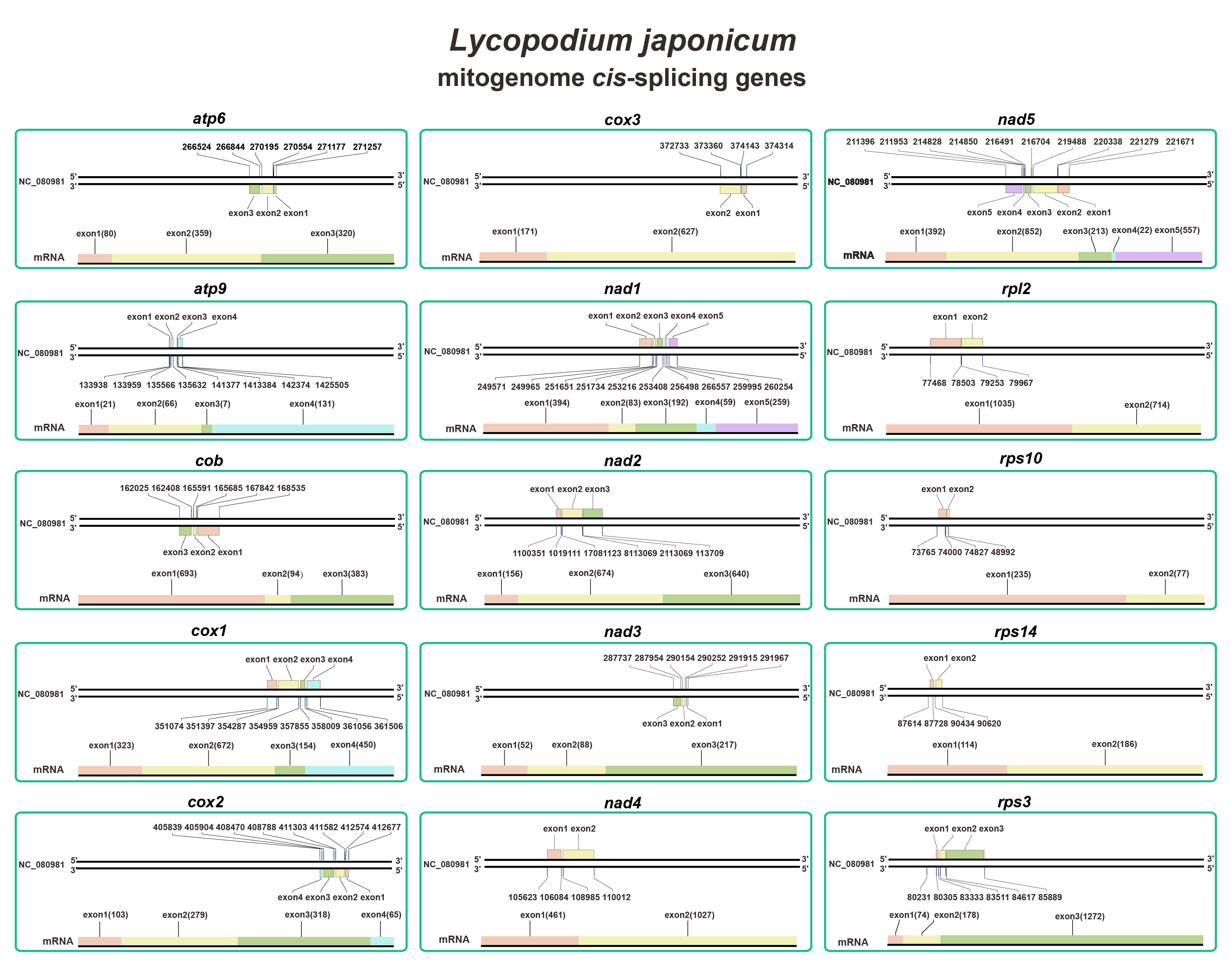

Supplement: Supplementary Figure 2 — The exon and intron contents of the Lycopodium japonicum mitogenome. [file Image2.jpeg]
